# Attitudes and perceptions of parents towards child and adolescent psychiatric consultation, diagnosis and treatment

**DOI:** 10.1192/bjo.2021.700

**Published:** 2021-06-18

**Authors:** Darpan Kaur, Rishab Verma, Rakesh Ghildiyal

**Affiliations:** Department of Psychiatry, Mahatma Gandhi Missions Medical College and Hospital, Sector 01, Kamothe, Navi Mumbai, Maharashtra, India

## Abstract

**Aims:**

The aim of the study was to assess the attitudes and perceptions of parents towards child and adolescent psychiatric consultation, diagnosis and treatment.

The hypothesis of the study was there are significant problems in the domains of attitude and perceptions of parents towards child and adolescent psychiatric consultation, diagnosis and treatment.

**Background:**

Parents are an important stake holder in child and adolescent psychiatry and mental health care service models. There is scarce literature from developing countries regarding attitudes and perceptions of parents towards child and adolescent psychiatric consultation, diagnosis and treatment.

**Method:**

This study was conducted at the Child and Adolescent Psychiatry Clinic, Department of Psychiatry at a Tertiary Care Institution. Eligibility criteria comprised of parents of children and adolescents who had come for consultation. The parents were provided information of the study and those willing to participate were included in the study. A convenience sample of 100 parents was considered for the study. The parents were interviewed using a specially designed survey comprising 30 questions with Yes/No response developed by the authors for the purpose of the study. Informed consent and Institutional Ethics Committee Clearance was obtained. Data were analysed using SPSS.

**Result:**

We found that the majority of parents were from urban area (72%) and mothers comprised 68%. We found that 46% of parents did not want a psychiatric diagnosis and 35 % parents felt stigmatized for seeing a psychiatrist for their child. Sixty nine percentage of parents preferred counseling as the first line of treatment and 31% preferred medicines as the first line of treatment. We found that 33 % felt additional psychological tests could be useful and 54% of parents felt brain imaging and blood tests could be useful for their child. Majority of parents expected basic improvement for their child within 1 week(32%) and expected full improvement by 1 month(82%). Fifty three percent of parents had searched online information prior to consulting and found useful information. However, 38% of the parents felt confused after reading online information and 69 % of parents were more worried about giving medications after referring online information.

**Conclusion:**

Our study provides useful key insights from parent's perspective in child and adolescent psychiatric services. Implications exist for future research as well as policy perspectives on the role, attitudes and expectations of parents as vital stake holders in child and adolescent psychiatry.

